# Centrifugal force assessment in ram sperm: identifying species-specific impact

**DOI:** 10.1186/s13028-021-00609-8

**Published:** 2021-11-04

**Authors:** Marta Neila-Montero, Marta F. Riesco, Mercedes Alvarez, Rafael Montes-Garrido, Juan Carlos Boixo, Paulino de Paz, Luis Anel-Lopez, Luis Anel

**Affiliations:** 1 Itra-ULE, INDEGSAL, University of León, Campus de Vegazana s/n, 24071 León, Spain; 2 Animal Reproduction and Obstetrics, Department of Veterinary Medicine, Surgery and Anatomy, University of León, Campus de Vegazana s/n, 24071 León, Spain; 3 Cellular Biology, Department of Molecular Biology, University of León, Campus de Vegazana s/n, 24071 León, Spain; 4 Anatomy, Department of Veterinary Medicine, Surgery and Anatomy, University of León, Campus de Vegazana s/n, 24071 León, Spain

**Keywords:** Centrifugation, Ejaculate handling, Ovine, Semen

## Abstract

**Background:**

Centrifugation is routinely employed in handling the ejaculates of some species, but it is not part of the commonly used protocols in ram. However, the development and implementation of new assisted reproductive technologies, alternative preservation models based on washing sperm from a cellular ageing-accelerating substance such as the seminal plasma, and basic studies in spermatology is associated with the use of centrifugation. This requires a specific evaluation of the centrifugation protocols considering the species-specific relationship with the potential damage produced by this procedure. No previous studies have determined the effect of different centrifugation forces on ram sperm. Therefore, we aimed to assess the performance of three centrifugal forces (600×*g*, 3000×*g*, and 6000×*g* for 10 min at room temperature) and their effects on ram sperm motility and functionality.

**Results:**

Sperm motility and functionality parameters were assessed at 0 h and after 2 h of incubation at 37 °C. As expected, a higher cell packaging degree was obtained at high centrifugation forces (P ≤ 0.0001). Cell packaging was unstable at all centrifugal forces. Thus, there was a high cell resuspension rate after less than 2 min. Regarding sperm quality, there was a change in movement pattern of 3000×*g* and 6000×*g* centrifuged sperm after 2 h of incubation at 37 °C, characterized by an increase in rapid progressive motility, linearity, straightness, and beat frequency, and a decrease in medium progressive motility, curvilinear velocity, path velocity, and head lateral amplitude. Non-significant differences were obtained among the different treatments concerning the total viability. However, we observed a significant increase (P ≤ 0.05) in the percentage of viable apoptotic sperm in the samples centrifuged at 6000×*g* at 0 h.

**Conclusions:**

Centrifugal forces equal to or greater than 3000×*g* induced some deleterious effects in ram sperm quality, and lower forces did not provide a successful cell packaging degree.

**Supplementary Information:**

The online version contains supplementary material available at 10.1186/s13028-021-00609-8.

## Background

Sperm centrifugation is a useful technique for many ejaculate handling procedures in several species. It is routinely performed in species with a low sperm concentration in ejaculates, such as humans [1], stallions [2], and boars [3], to provide a suitable volume of the sperm dose required for artificial insemination. It is also performed in goats to separate sperm from the seminal plasma, since seminal plasma contains a phospholipase that can hydrolyze the membrane phospholipids of sperm when they are subjected to freezing [4, 5]. The development and implementation of advanced assisted reproductive techniques, news alternatives in sperm preservation or evaluation protocols, would extend the spectrum of target species using centrifugation protocols. In fact, this procedure can be used in all species as a sperm selection method for the isolation of morphologically normal and motile sperm, improving the sperm quality sample [6, 7], or for the separation of sperm containing X and Y chromosomes [8–10]. In addition, researchers have recently suggested that the removal of seminal plasma may be beneficial for sperm preservation in most farm animals [11]. Prolonged exposure of sperm to the seminal plasma during liquid in vitro storage creates an unphysiological situation because after natural mating sperm are quickly separated from the seminal plasma in the female tract [12]. Thus, the separation of sperm from the seminal plasma with subsequent resuspension in a culture medium is becoming an increasingly regular practice in the field of assisted mammalian reproduction [13]. Centrifugation, therefore, is one of the most common procedures in ejaculate handling. Nevertheless, this washing procedure not only complicates semen processing but can also be a potentially sperm-damaging step [14, 15]. The main objective of centrifuging semen is removing the liquid fraction and recovering the maximum number of sperm from the initial population with minimum adverse effects on their function [6, 16]. However, centrifugation has two problems that are closely linked: (1) the sperm loss due to the removal of the supernatant [17], and (2) the physical damage caused to the sperm population [1]. In mouse [17], human [18–20], dog [21], stallion [22–24], and boar [3, 16], it has been proven that the centrifugation regime—centrifugation force and duration—influences both the sperm recovery and yield in a similar fashion. Using lower centrifugal forces and shorter centrifugation times leads to lower sperm recovery due to a lack of complete pelleting and, consequently, the loss of unpelleted cells upon supernatant removal [3, 16, 17]. In contrast, using higher centrifugal forces and longer centrifugation times leads to greater sperm sedimentation but has a deleterious effect on sperm reducing their motility and viability [18–24]. Therefore, the centrifugation regime must achieve a balance between the centrifugation performance (here evaluated by cell packaging degree) and its effect on sperm quality. In this sense, species specificity is very important to sperm injury caused by centrifugation [16]. In some mammals, such as rodents [17, 25–27], dogs [21] and humans [1, 28–31], sperm have been shown to be very sensitive to mechanical and centrifugal forces, and must be manipulated under carefully defined conditions to reduce sperm damage (200–400×*g* for 5 to 12 min in rodents [17], 720×*g* for 5 min in dogs [21], and 300–600×*g* for 10–20 min in humans [19, 20]). In any case, all studies recommend the use of centrifugal forces below of 800×*g*. Sperm from other species, such as equine [32–34], bovine [33, 35], caprine [5], porcine [3, 16], and bear [36], are somewhat less sensitive to centrifugation, which allows the use of centrifugal forces up to 2400×*g* for 5 min in stallion [24], 5000×*g* for 5 min in bull [7], 3000×*g* for 3 min in goat [37], 2400×*g* for 3 min in boar [16], and 9600×*g* for 6 min in brown bear [36]. With regard to ram sperm, the few studies evaluating the effects of centrifugation were conducted in the 1990s. At the time when these studies were conducted, the protocol for freezing ram semen in Sweden and Norway included the concentration of previously extended sperm by centrifugation at 700×*g* for 10 min. Although centrifugation was rejected by one study as being harmful to ram sperm [38], another study demonstrated that the final number of sperm decreased substantially by removing the supernatant after centrifugation [39].

Based on the published research, the effects of centrifugation on sperm seem to be species-specific, and few studies have been carried out in ram. Thus, the objectives of this work were to assess the performance of three centrifugal forces (600×*g*, 3000×*g*, and 6000×*g* for 10 min at room temperature) and their effects on ram sperm quality. We aimed to identify the species-specific problems of ram sperm centrifugation.

## Methods

### Reagents and media

The Zombie Violet™ Fixable Viability Kit was purchased from BioLegend (Saint Diego, CA, USA), whereas the CellEvent™ Caspase-3/7 Green Detection Reagent and CellROX™ Deep Red Reagent were acquired from Thermofisher (Waltham, MA, USA). Lyophilized Zombie Violet™ dye was reconstituted in Dimethyl Sulfoxide (DMSO) following the manufacturer’s instructions (100 µL of DMSO to one vial of Zombie Violet™ dye). CellEvent™ Caspase-3/7 and CellROX™ Deep Red were purchased as 2 mM and 2.5 mM stabilized solutions, respectively. Stock solutions of fluorescence probes were prepared at 1 µL and kept at − 20 °C in the dark until needed.

Other chemicals, such as extender components, were supplied by Sigma (Saint Louis, MI, USA).

### Animals

In total, 11 adult rams (ranging from 2–9 years old) of the Assaf breed, of proven fertility, and trained for semen collection on an artificial vagina on a regular basis (two collections 2 days per week) were used. Animals were housed and fed a standard balanced diet at the Animal Selection and Reproduction Centre of the Junta de Castilla y León (CENSYRA; Villaquilambre, León, Spain). The animal manipulations were performed in accordance with the European Union Regulation 2010/63/EU. All experiments were approved by the Ethics Committee for Experimentation with Animals of the University of León (ÉTICA-ULE-013-2018).

### Study design

Our experimental design attempted to restrictively evaluate the effects of centrifugation in ram sperm without considering temperature and storage time. In this sense, semen was collected during April and May. One ejaculate per male was collected by artificial vagina at 40 °C (IMV Technologies, L’Aigle, France) in the presence of a female teaser. A preliminary semen evaluation was carried out immediately after the collection to select samples. This evaluation included a determination of: (1) ejaculate volume by collecting them in graduated tubes, (2) mass motility (0–5) assessing a 5 μL drop at 40× in a microscope equipped with a warmed stage programmed at 37 °C (Leica DM LB, Meyer Instruments, Houston, TX, USA), and (3) sperm concentration by a cell counter (NucleoCounter SP-100, ChemoMetec, Allerod, Denmark). In total, 11 ejaculates that met the following conditions were considered valid: volume ≥ 0.5 mL, mass motility ≥ 3, and concentration ≥ 3000 × 10^6^ sperm/mL. Ejaculates were individually diluted at a ratio of 1:2 (v/v) in a TES-Tris-Fructose medium supplemented with 20% egg yolk prepared in the manner previously described by our research group [40] (300 mOsm/kg, pH 7.2), and transported to the laboratory at 35 °C in a water bath. At that point, samples were divided into four aliquots of the same volume, which were treated: without centrifugation (Control) and with 600×*g*, 3000×*g*, or 6000×*g* for 10 min at room temperature. After that, the cell packaging degree of the centrifuged samples was assessed, and pellets were resuspended using a pipette with cut end tips. At that time, a first evaluation of the sperm samples was carried out, performing a second evaluation after submitting the samples to a thermal stress test by incubating them at 37 °C in an incubator (Incucell V 55, MMM Medcenter, Planegg, Germany) for 2 h. The last trial was performed to evidence the sublethal damages that could remain undetectable immediately after centrifugation.

### Technique performance evaluation

The performance of the centrifugation was determined by assessing the cell packaging degree. The cell packaging degree of the centrifuged samples was first subjectively assessed (subjective pelletization degree, 0 to 5 score), assigning a value of 0 to the samples in which a pellet was not observed and 5 when a well-defined pellet was visualized. In a second assay, the cell packaging degree was indirectly assessed by removing 10 µL on the top of the surface supernatant immediately after centrifugation (0 min) and after 2 min with the aim of evaluating the stability of the pellet. Then, the 10 µL of surface supernatant was mixed with 10 µL of 2% glutaraldehyde fixative solution (1% final concentration, v/v), prepared in the manner previously described by our research group [40], to determine the sperm concentration using a Makler counting cell chamber (10 μm depth; Sefi Medical Instruments, Haifa, Israel) with a Computer-Aided Sperm Analysis (CASA) system (Sperm Class Analyzer—SCA—6.3.0.59; Microptic S.L., Barcelona, Spain).

### Semen evaluation

#### Motility and kinetic parameters by a CASA system

The motility and kinetic parameters were assessed using the CASA system (Sperm Class Analyzer—SCA—6.3.0.59; Microptic S.L., Barcelona, Spain) set to capture a total of 50 frames at 100 frames/second and particles with an area of 20–70 μm^2^. Aliquots of each treatment were diluted to 25 × 10^6^ sperm/mL in phosphate-buffered saline (PBS; 300 mOsm/kg, pH 7.2) and tempered on a 37 °C plate for 5 min. After that, a 5 μL drop was placed in a Makler counting cell chamber. Samples were examined with a 10× negative phase contrast objective in a microscope equipped with a warmed stage at 38 °C (Eclipse E400, Nikon, Tokyo, Japan). At least 400 sperm from 4 different randomly selected fields were captured and analyzed. The reported kinetic parameters were: curvilinear velocity (VCL, μm/s); velocity according to the straight path (VSL, μm/s); velocity according to the smoothed path (VAP, μm/s); linearity (LIN, %); straightness (STR, %); wobble (WOB, %); amplitude of the lateral displacement of the sperm head (ALH, μm); head beat-cross frequency (BCF, Hz); total motility (TM), defined as the percentage of sperm with VCL > 15 μm/s; progressive motility (PM), defined as the percentage of sperm with VCL > 45 μm/s; rapid progressive motility (RAP PM), defined as the percentage of sperm with VCL > 75 μm/s; and medium progressive motility (MED PM), defined as the percentage of sperm with VCL 45–75 μm/s.

#### Cellular functionality by flow cytometry

##### Staining for determination of viability, caspases 3 and 7 activity, and mitochondrial functionality

The combination Zombie Violet™ Fixable Viability Kit (excitation 405 nm, emission 423 nm), CellEvent™ Caspase-3/7 Green Detection Reagent (excitation 502 nm, emission 530 nm), and CellROX™ Deep Red (excitation 644 nm, emission 655 nm) was used to simultaneously determine viability through plasma membrane integrity, caspases 3 and 7 activation as a marker of apoptosis, and mitochondrial function through reactive oxygen species (ROS) content, respectively.

The Zombie Violet™ Fixable Viability Kit is an amine-reactive fluorescent dye that is non-permeant to live cells but permeant to the cells with compromised membranes. Thus, it can be used to assess the live and dead status of mammalian cells by determining two subpopulations: (1) the subpopulation with intact membranes (viable sperm low stained by Zombie Violet™), and (2) the subpopulation showing compromised membranes (dead sperm high stained by Zombie Violet™).

The CellEvent™ Caspase-3/7 Green Detection Reagent is a cell-permeant reagent that consists of a four-amino acid peptide (DEVD) conjugated to a nucleic acid-binding dye. During apoptosis, caspases 3 and 7 proteins are activated and able to cleave the caspase 3/7 recognition sequence encoded in the DEVD peptide. Cleavage of the recognition sequence and binding of the DNA by the reagent labels the apoptotic cells with a bright fluorogenic signal, producing two cellular subgroups: (1) the subgroup with inactive caspases 3 and 7 (non-apoptotic sperm non-stained by CellEvent™ Caspase-3/7), and (2) the subgroup with active caspases 3 and 7 (apoptotic sperm stained by CellEvent™ Caspase-3/7).

CellROX™ Deep Red is also a cell-permeant reagent that has been developed for the detection and quantitation of ROS (mainly superoxide anion) in the cytoplasm of live cells. This dye is non-fluorescent or very weakly fluorescent in the reduced state. Upon oxidation, the reagent exhibits strong fluorescence and remains localized within the cell. This makes it possible to identify two cell types: (1) sperm with low superoxide anion content (sperm with low mitochondrial functionality non-stained by CellROX™), and (2) sperm with high superoxide anion content (sperm with intense mitochondrial activity stained by CellROX™).

For staining, we adopted a protocol previously described by Riesco et al. [41]. Briefly, the Zombie Violet™ stock solutions were resuspended in 1 mL of PBS, while CellEvent™ Caspase-3/7 and CellROX™ were resuspended in 10 μL of PBS. Samples were diluted in PBS to a concentration of 2 × 10^6^ sperm/mL, and the cells were washed by short centrifugation (15″; MiniSpin plus, Eppendorf, Hamburg, Germany) with the removal of the supernatant. Then, cells were incubated at room temperature in the dark for 30 min with 96 μL Zombie Violet™, 2 μL CellEvent™ Caspase-3/7, and 2 μL CellROX™. After that, a new wash was performed to stop cell staining and avoid an overstaining effect. Then, the pellet was resuspended in 1 mL PBS, and the flow cytometry analysis was immediately conducted.

##### Flow cytometry analyses

Flow cytometry analyses were conducted in a MACSQuant Analyzer 10 (Miltenyi Biotech, Bergisch Gladbach, Germany), equipped with three lasers emitting at 405 nm, 488 nm, and 635 nm (violet, blue, and red, respectively) and 10 photomultiplier tubes. Violet fluorescence was detected in V1 (excitation 405 nm, emission 450/50 nm), green fluorescence was detected in B1 (excitation 488 nm, emission 525/50 nm), and red fluorescence was detected in R1 [excitation 635 nm, emission 655–730 nm (655LP + split 730)]. Samples were acquired using MACS Quantify software (Miltenyi Biotech, Bergisch Gladbach, Germany) recording a total of 40,000 cells per sample at a flow rate of 200–300 cells/s.

The instrument was calibrated before starting the experiment using the specific calibration beads provided by the manufacturer. Compensation for the spectral overlap was not necessary because the flow cytometer have laser spatially separated [42]. Data were analyzed using FlowJo V 10.2 (Ashland, OR, USA). Representative cytograms of the assay and gating strategy are shown in Additional file 1. Non-sperm events (debris) and doubles were identified and eliminated from the analysis (Additional file 1 a, b), and unstained controls were used to determine the positive and negative events (Additional file 1 c, e). Subpopulations were divided by quadrants, and the frequency of each subpopulation was quantified (Additional file 1 d, f).

### Statistical analysis

The SAS/STAT^®^ V 9.1 statistical package (SAS Institute, Cary, NC, USA) was used for data analysis. The normality of variables was examined. Normally distributed data were analyzed by a mixed linear model (MIXED procedure). Subjective pelletization degree was analysed by Kruskal–Wallis test (NPAR1WAY procedure). The centrifugal forces were included as the main effects, and the male was recorded as a random effect in the model. The differences between the LS-means (Least Squares Means) were computed by Dunnett’s test. The performance of the centrifugation was studied by Pearson’s correlations. The results are displayed as means ± SEM (Standard Error of the Mean), and the threshold for significance was P ≤ 0.05.

## Results

### Technique performance evaluation

**Fig. 1:**
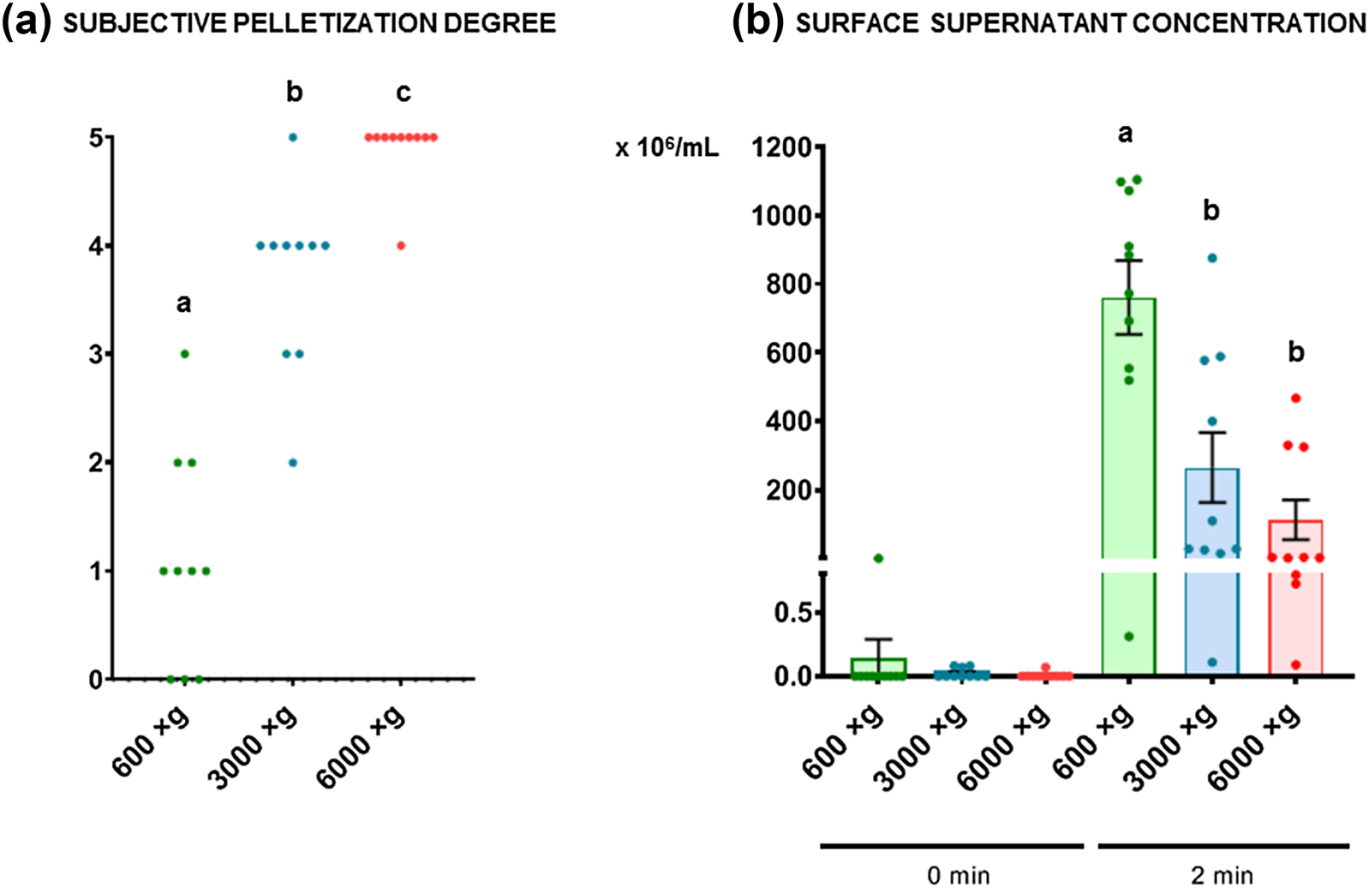
Performance of three centrifugal forces on ram sperm. **a** Subjective pelletization degree (0–5 score); **b** Surface supernatant concentration on the top immediately after centrifugation (0 min) and after 2 min (× 10^6^ sperm/mL). The same 10 males were analyzed in each experimental group (600×*g*, 3000×*g*, and 6000×*g* for 10 min at room temperature). Graph dots represent individual male ejaculates. Different lowercase superscripts letters (**a**, **b**, **c**) indicate significant differences (P ≤ 0.0001) for each parameter and assessment point among the centrifugal forces

A Pearson’s correlation study was performed to evaluate the relationship among the cell packaging degree, sperm mass motility, and sperm concentration of the individual ejaculates. Non-significant correlations were found among these variables (P > 0.05), obtaining Pearson’s correlation coefficients (r) lower than ± 0.59 in all cases. The subjective pelletization degree increased significantly (P ≤ 0.0001) when the centrifugation force increased (Fig. 1a). The subjective pelletization degree was optimal (value of five) at 6000×*g* for practically all the males analyzed (Fig. 1a). The surface supernatant concentration value at 0 min was around 0 in all centrifugal forces (P > 0.05). However, at 2 min, that concentration increased greatly for all treatments, being significantly higher (P ≤ 0.0001) at 600×*g* than at the other centrifugal forces (Fig. 1b).

### Semen evaluation

#### Motility and kinetic parameters

**Fig. 2:**
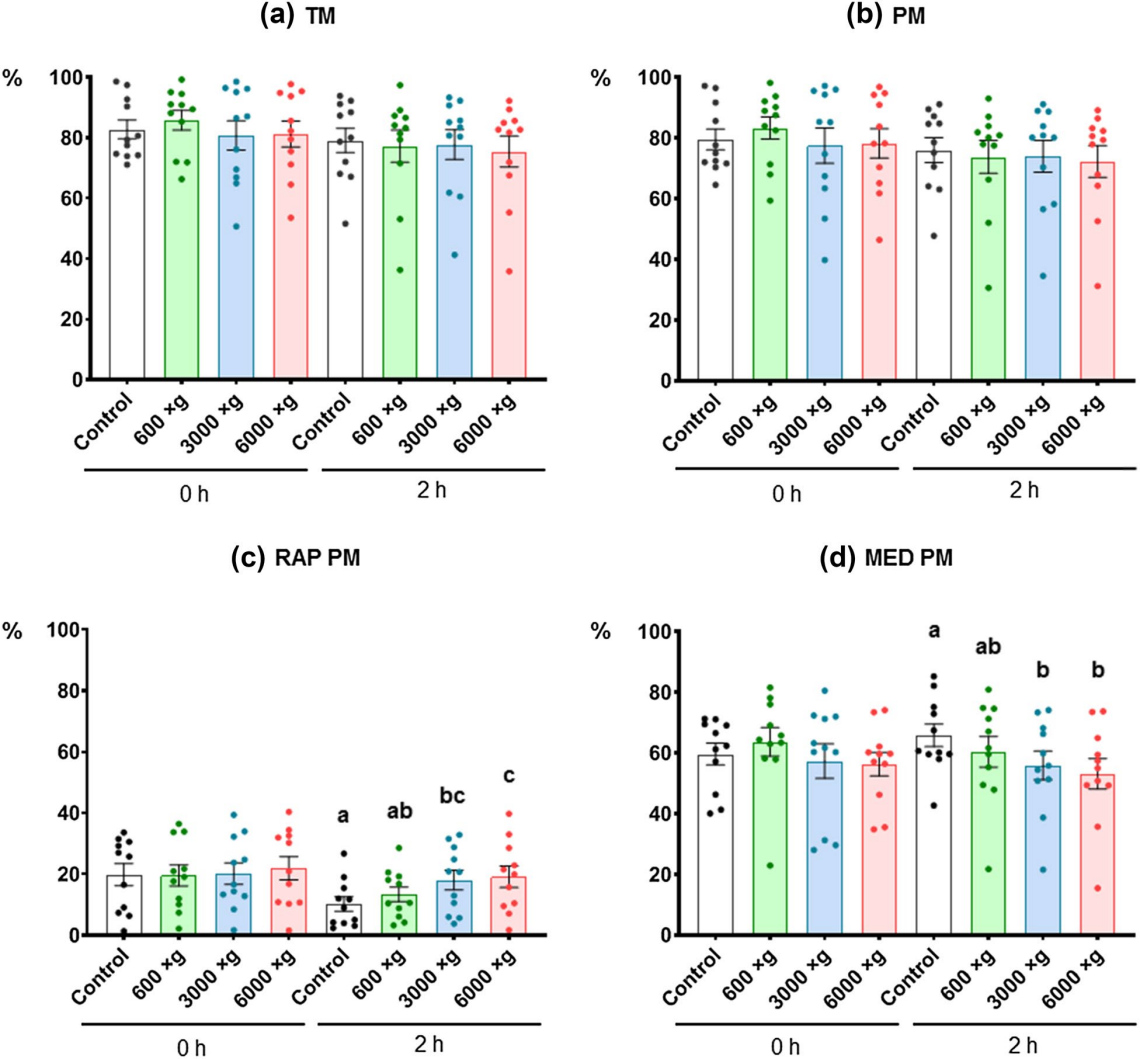
Effect on ram sperm motility induced by three centrifugal forces. **a** TM, total motility (%); **b** PM, progressive motility (%); **c** RAP PM, rapid progressive motility (%); **d** MED PM, medium progressive motility (%). The same 11 males were analyzed in each experimental group (600×*g*, 3000×*g*, and 6000×*g* for 10 min at room temperature) at 0 h and 2 h post-incubation at 37 °C. Graph dots represent individual male ejaculates. Different lowercase superscripts letters (**a**, **b**, **c**) indicate significant differences (P ≤ 0.05) for each assessment point among the centrifugal forces

The obtained TM and PM percentages were high (> 80% and > 77%, respectively) in all treatments at the initial time (0 h) (P > 0.05). After 2 h of incubation, non-significant differences were found among the different treatments (P > 0.05) (Figs. 2a, b). However, when the subpopulation of rapid progressive sperm (RAP PM) was analyzed after 2 h of incubation at 37 °C, a significantly higher percentage (P ≤ 0.05) was obtained in the samples centrifuged at the highest centrifugal forces (3000×*g* and 6000×*g*), with non-significant differences between both forces (P > 0.05) (Fig. 2c). We similarly observed that the subpopulation of medium progressive sperm (MED PM) decreased significantly (P ≤ 0.05) in the same treatments and at the same timepoints (Fig. 2d). With respect to the sperm kinematics, the VCL, VAP, and ALH decreased significantly at 2 h (P ≤ 0.05) in the samples centrifuged at 3000×*g* and 6000×*g* (Table 1). The LIN, STR, and BCF were all significantly higher (P ≤ 0.05) at 2 h in the samples centrifuged at 3000×*g* and 6000×*g*, with non-significant differences (P > 0.05) observed at the initial time (0 h) (Table 1).

**Table 1:** Effect on ram sperm kinetic parameters induced by three centrifugal forces

Parameter	Control	600×*g*	3000×*g*	6000×*g*
***VCL (µm/s)***				
0 h	296.6 ± 17.9^ab^	307.9 ± 16.0^a^	289.0 ± 16.7^ab^	277.9 ± 8.9^b^
2 h	318.3 ± 14.8^a^	307.5 ± 14.0^a^	284.5 ± 13.9^b^	274.1 ± 10.3^b^
***VSL (µm/s)***				
0 h	89.7 ± 4.6^ab^	93.9 ± 4.5^a^	87.7 ± 5.5^ab^	86.8 ± 4.8^b^
2 h	80.4 ± 3.0	83.7 ± 3.4	81.4 ± 4.5	81.6 ± 5.1
***VAP (µm/s)***				
0 h	148.7 ± 6.0^ab^	153.5 ± 5.5^a^	142.9 ± 6.7^ab^	141.6 ± 3.3^b^
2 h	152.2 ± 5.1^a^	145.9 ± 4.9^a^	135.7 ± 4.7^b^	134.0 ± 3.2^b^
***LIN (%)***				
0 h	31.8 ± 2.1	31.6 ± 1.9	30.7 ± 1.9	31.8 ± 1.8
2 h	26.6 ± 1.4^a^	28.0 ± 1.5^ab^	29.2 ± 1.6^b^	30.1 ± 1.8^b^
***STR (%)***				
0 h	59.9 ± 3.2	60.1 ± 2.6	59.2 ± 3.0	59.7 ± 3.0
2 h	52.7 ± 2.1^a^	56.3 ± 2.6^ab^	57.7 ± 2.7^bc^	58.8 ± 3.2^bc^
***WOB (%)***				
0 h	52.0 ± 1.3	51.2 ± 1.2	50.3 ± 1.1	51.9 ± 1.0
2 h	49.4 ± 0.8^ab^	48.6 ± 0.7^a^	49.0 ± 0.9^ab^	50.1 ± 1.0^b^
***ALH (µm)***				
0 h	4.1 ± 0.2^ab^	4.2 ± 0.2^a^	3.9 ± 0.2^ab^	3.8 ± 0.1^b^
2 h	4.3 ± 0.2^a^	4.2 ± 0.2^a^	3.9 ± 0.2^b^	3.7 ± 0.1^b^
***BCF (Hz)***				
0 h	24.4 ± 1.4	25.9 ± 1.6	26.2 ± 1.6	26.5 ± 1.4
2 h	22.5 ± 0.9^a^	22.9 ± 0.7^ab^	24.0 ± 0.8^bc^	24.8 ± 0.9^c^

The same 11 males were analyzed in each experimental group (600×*g*, 3000×*g*, and 6000×*g* for 10 min at room temperature) at 0 h and 2 h post-incubation at 37 °C. Different lowercase superscripts letters (a, b, c) indicate significant differences (P ≤ 0.05) for each parameter and assessment point among the centrifugal forces

VCL, curvilinear velocity (µm/s); VSL, straight line velocity (µm/s); VAP, path velocity (µm/s); LIN, linearity (%); STR, straightness (%); WOB, wobble (%); ALH, head lateral amplitude (µm); BCF, beat frequency (Hz)

#### Cellular viability, caspases 3 and 7 activity, and mitochondrial functionality

**Fig. 3:**
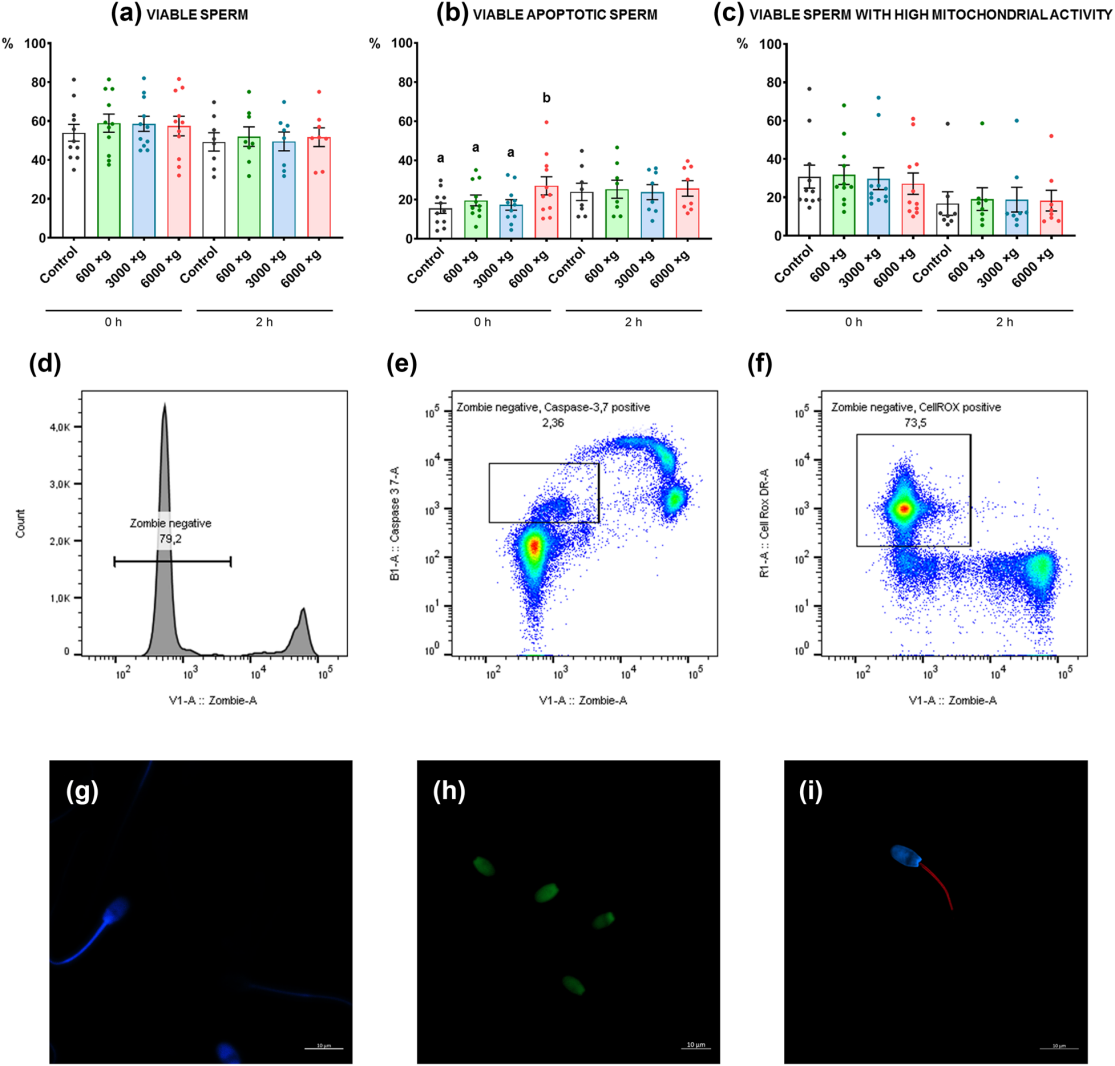
Effect on ram sperm functionality induced by three centrifugal forces. Ram sperm **a** viability (Zombie Violet™) (%), **b** caspases 3 and 7 activation (CellEvent™ Caspase-3/7 Green) (%), and **c** high mitochondrial activity (CellROX™ Deep Red) (%). The same 11–8 males were analyzed in each experimental group (600×*g*, 3000×*g*, and 6000×*g* for 10 min at room temperature) at 0 h and 2 h post-incubation at 37 °C. Graph dots represent individual male ejaculates. Different lowercase superscripts letters (**a**, **b**) indicate significant differences (P ≤ 0.05) for each assessment point among the centrifugal forces. FlowJo analyses for **d** viability (Zombie Violet™), **e** apoptosis (CellEvent™ Caspase-3/7 Green), and **f** high mitochondrial functionality (CellROX™ Deep Red). Ram sperm confocal images of Control samples labelling for **g** plasma membrane (Zombie Violet™), **h** DNA (CellEvent™ Caspase-3/7 Green), and **i** intracellular ROS (CellROX™ Deep Red) employed in the flow cytometry analyses

Immediately after centrifugation (0 h), viability and mitochondrial functionality were similar among the different treatments (P > 0.05) (Figs. 3a, c). A significant increase (P ≤ 0.05) was only observed in the percentage of viable apoptotic sperm, with caspases 3 and 7 being active, in the samples centrifuged at 6000×*g* (Fig. 3b). However, despite the absence of any significant differences in the percentage of viable sperm with high mitochondrial activity among the different treatments (P > 0.05), there was a decreasing trend (P = 0.1) when the centrifugal force increased from 600 to 6000×*g* (Fig. 3c). After 2 h of incubation, non-significant differences (P > 0.05) appeared in the sperm functionality parameters studied.

## Discussion

Centrifugation is one of the most common sperm preparation techniques for both experimental and practical programs [16]. However, understanding the centrifugation consequences on the recovered ram sperm quantity and quality requires an in depth analysis considering the previously described species-specific factors. Although sperm centrifugation is not a common practice in ram, the development and implementation of advanced procedures linked with centrifugation in ejaculate management, such a sperm selection, sperm sex-sorting and in vitro fertilization, new models of sperm preservation, and evaluation protocols, justifies the study of species-specific centrifugation protocols adapted to ram sperm characteristics.

In this study, we investigated the effects of three centrifugal forces (600×*g*, 3000×*g*, and 6000×*g* for 10 min at room temperature) on ram sperm motility and functionality, as well as their performance, by assessing the cell packaging degree obtained at each centrifugation force. Attending to our next objective, which will be consist on wash ram sperm to separate it from the seminal plasma, cell packaging degree should be optimal to lose the lowest number of sperm possible in the process without damaging the sperm quality. In a study recently conducted by Paul et al. [43] in ram sperm, seminal plasma was removed by a 1:15 dilution (200 × 10^6^ sperm/mL) and centrifugation at 150×*g* for 10 min. However, in this study, the analysis of the cell packaging degree, which is related to the percentage of cellular recovery obtained after the centrifugation and removal of seminal plasma, was not contemplated. Our group previously demonstrated the importance of evaluating this percentage in other species, such as brown bear. We observed that at low centrifugal forces, a large amount of sperm was lost during the centrifugation process [44]. Thus, for the first time, we considered the development of a centrifugation protocol for ram sperm with acceptable recovery rates without damaging sperm. First, a Pearson’s correlation study was performed to evaluate if the sperm mass motility or sperm concentration of the individual males affected the cell packaging degree. We hypothesized that a male-specific response could occur with respect to the centrifugation protocols. In particular, male-specific effects have been demonstrated in different processes, including freezability [45]. This hypothesis was discarded by obtaining non-significant Pearson’s correlation coefficients (r) lower than ± 0.59 in all cases (P > 0.05), which indicated that the cell packaging degree is not affected by the male factor. Then, we observed that the subjective pelletization degree significantly increased as the centrifugal force increased (P ≤ 0.0001), obtaining a maximum subjective pelletization degree at 6000×*g*. Regarding the indirect measurement of the cell packaging degree, the surface supernatant concentration at 0 min was around 0 in all centrifugal forces. However, at 2 min, the concentration increased considerably for all treatments, especially at 600×*g*, as expected (P ≤ 0.0001). This indicated that cell packaging was unstable, since it was possible to visualize how quickly the pellet dissolved quickly, likely due to the vigorous movement of the ram sperm, which was visible even macroscopically [46]. This hinders the handling of ejaculates in reproduction centers due to the short time available to achieve seminal plasma removal before pellet resuspension, which would result in considerable sperm loss. However, these parameters were not the only object of analysis of this work since a good balance is required between the percentage of sperm recovery after centrifugation and the quality of the recovered sample.

Concerning the sperm quality analysis, our results showed a change in the movement pattern of centrifuged sperm at 3000×*g* and 6000×*g* after 2 h of incubation, which was characterized by an increase in the RAP PM, LIN, STR, and BCF, and a decrease in the MED PM, VCL, VAP, and ALH. Traditionally, the high VCL and ALH and the low LIN or WOB are used as kinematic markers of the hyperactivation state, which is determined by changes in the flagellar flap pattern, although the trends observed in that state are opposite to those obtained in this work [47, 48]. This hyperactivation has been used as a marker of the state of sperm capacitation in several mammal species [49], such as hamster [50], guinea pig [51, 52], mouse [53], dog [54], rabbit [55], bat [56], dolphin [57], sheep [58], and human [59]. Therefore, it could be hypothesized that high centrifugal forces could inhibit the hyperactivation and capacitation abilities of sperm, which appear to have physiological relevance in fertilization since they have been observed in or near the oviduct portion where fertilization occurs [47]. This hypothesis is partially supported by the increase in the RAP PM at both centrifugal forces, since the non-hyperactive and progressive trajectories are equivalent, according to previous studies in different species [47]. Hyperactivated sperm often move in large circles due to the asymmetric flagellar flap pattern [59], therefore reducing their progressive movement. Further studies to analyze sperm capacitation at these high centrifugal forces will be necessary to demonstrate this hypothesis.

At the level of cellular functionality, numerous flow cytometry protocols have been developed in the last decade which have allowed the simultaneous assessment of multiple parameters related to the sperm quality and function by combining different probes [60, 61]. Sperm membrane integrity is one of the key assays in sperm evaluation since the sperm plasma membrane is an important structure that protects sperm against extracellular injuries and adapts the sperm to a variety of physiological and environmental challenges, including capacitation and acrosomal exocytosis [62, 63]. The use of the Zombie Violet™ Fixable Viability Kit allowed us to identify viable sperm through the physical integrity of the membrane, and non-differences were observed concerning the total viability among the different treatments (P > 0.05). However, a good sperm washing method should allow the recovery of the maximum number of cells by modifying the physiological status of the sperm as little as possible. The protocol must be optimized so that motility and viability are not altered, while considering the importance of other parameters in sperm preservation, such as the capacitation state and premature senescence due to apoptotic changes, which can compromise the sperm’s ability to fertilize the oocytes [64, 65]. Apoptosis is an important process in the development of male germ cells, since it organizes their production and functionality from the early stages of gonadal differentiation until the moment of fertilization [66]. In sperm, apoptosis can be activated as a mechanism of elimination of abnormal or senescent sperm during normal spermatogenesis and in response to environmental stress or injury [13]. Apoptosis can be initiated by stimuli from outside or inside of the sperm, such as DNA damage or loss of the mitochondrial membrane potential [13, 67]. In addition, altered apoptotic processes have been observed to be closely associated with male infertility [68, 69]. Assays used to detect apoptosis in sperm include recognizing the activated caspases [61], as in the case of the CellEvent™ Caspase-3/7 Green Detection Reagent. Therefore, it was necessary to conduct a more exhaustive analysis, to find significant differences (P ≤ 0.05) among the different treatments concerning apoptosis. Caspases 3 and 7, specific cysteinyl aspartate proteases that execute the breakdown of structural proteins and DNA [13], were activated in a higher percentage of sperm in the centrifuged samples at 6000×*g* at 0 h (P ≤ 0.05), and did not produce significant differences (P > 0.05) at 2 h of incubation. The sperm samples after 2 h of incubation suffered significant quality damage that masked the treatment effect. Therefore, significant differences among the experimental groups were not observed. Finally, the use of CellROX™ Deep Red was also interesting, since sperm mitochondria are sensitive indicators of sperm stress [70, 71]. ROS, and specifically superoxide anion content, did not show significant differences (P > 0.05) among the different treatments. However, a trend was observed at 0 h in which, at a greater centrifugation force, the subpopulation of sperm with intense superoxide anion content decreased within the population of viable sperm. This could indicate a negative effect because the superoxide anion production may reflect intense mitochondrial activity rather than oxidative stress [42]. In stallions, the strongest sperm have an intense mitochondrial activity [72] because mitochondria are the epicenter of the modulation of the vast majority of processes that allow the sperm to remain in an optimal state [73]. ROS localization is another key factor. ROS can be colocalized with the mitochondria, but can also be found in the nucleus as a potential inductor of DNA damage [74]. In our study, ROS distribution was studied by confocal microscopy, only confirming the staining of the middle piece (Fig. 3i). For this, we concluded that high centrifugal forces reduced the mitochondrial activity of ram sperm, and we assumed that this was a negative effect.

## Conclusions

The cell packaging degree improved significantly at high centrifugal forces (3000×*g* and 6000×*g*). However, these two centrifugal forces altered the sperm movement pattern after 2 h of incubation at 37 °C by increasing the RAP PM, LIN, STR, and BCF and decreasing the MED PM, VCL, VAP, and ALH. The apoptosis process (caspases 3 and 7) was activated in a higher percentage of sperm in the centrifuged samples at 6000×*g* at 0 h. Thus, centrifugal forces equal to or greater than 3000×*g* induced some deleterious effects on ram sperm functionality.

## Supplementary Information

**Additional file 1.** Representative cytograms of the assays reported in the present study. **(a)** Dot plot showing the region gated corresponds to sperm. **(b)** Events gated in **(a)** are now plotted against SSC-H and SSC-A for select single cells, and this region was further used to set the remaining populations of interest. **(c)** Unstained control for viability and apoptosis. **(d)** Representative dot plot confronting Zombie Violet™ (X axis) versus CellEvent™ Caspase-3/7 Green fluorescence (Y axis), which evidences three subpopulations: (1) viable non-apoptotic sperm negative for Zombie Violet™ and CellEvent™ Caspase-3/7 Green, (2) viable apoptotic sperm negative for Zombie Violet™ and positive for CellEvent™ Caspase-3/7 Green (subpopulation of interest), and (3) dead sperm positive for Zombie Violet™ and CellEvent™ Caspase-3/7 Green. **(e)** Unstained control for viability and mitochondrial functionality. **(f)** Representative cytogram showing relation between Zombie Violet™ (X axis) and CellROX™ Deep Red (Y axis) which allowed us to detect three subpopulations: (1) viable sperm with low mitochondrial activity negative for Zombie Violet™ and CellROX™ Deep Red, (2) viable sperm with high mitochondrial activity negative for Zombie Violet™ and positive for CellROX™ Deep Red (subpopulation of interest), and (3) dead sperm positive for Zombie Violet™ and CellROX™ Deep Red.

## Data Availability

The datasets used and/or analysed during the current study are available from the corresponding author on reasonable request.

## Declarations

### Prior publication

Data included in this article have previously been published in the 15º Congreso Internacional de la Asociación Española de Reproducción Animal, Toledo, España, November 7^–^9th 2019.
