# Supplementary material for: Centrifugal force assessment in ram sperm: identifying species-specific impact
Source: Acta Vet Scand. 2021 Nov 4;63:42. doi: 10.1186/s13028-021-00609-8 (PMC8567708; doi:10.1186/s13028-021-00609-8)
Supplement: Supplementary file 1 — Additional file 1. Representative cytograms of the assays reported in the present study. (a) Dot plot showing the region gated corresponds to sperm. (b) Events gated in (a) are now plotted against SSC-H and SSC-A for select single cells, and this region was further used to set the remaining populations of interest. (c) Unstained control for viability and apoptosis. (d) Representative dot plot confronting Zombie Violet™ (X axis) versus CellEvent™ Caspase-3/7 Green fluorescence (Y axis), which evidences three subpopulations: (1) viable non-apoptotic sperm negative for Zombie Violet™ and CellEvent™ Caspase-3/7 Green, (2) viable apoptotic sperm negative for Zombie Violet™ and positive for CellEvent™ Caspase-3/7 Green (subpopulation of interest), and (3) dead sperm positive for Zombie Violet™ and CellEvent™ Caspase-3/7 Green. (e) Unstained control for viability and mitochondrial functionality. (f) Representative cytogram showing relation between Zombie Violet™ (X axis) and CellROX™ Deep Red (Y axis) which allowed us to detect three subpopulations: (1) viable sperm with low mitochondrial activity negative for Zombie Violet™ and CellROX™ Deep Red, (2) viable sperm with high mitochondrial activity negative for Zombie Violet™ and positive for CellROX™ Deep Red (subpopulation of interest), and (3) dead sperm positive for Zombie Violet™ and CellROX™ Deep Red. [file 13028_2021_609_MOESM1_ESM.pdf]

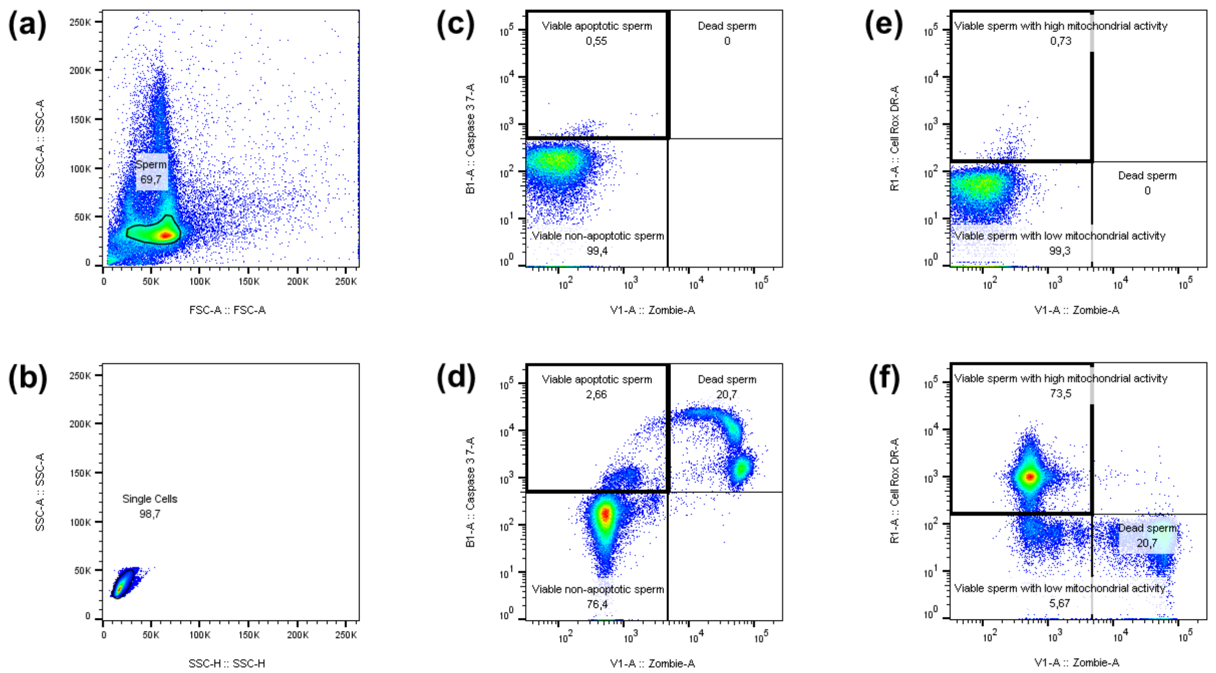

Additional file 1. Representative cytograms of the assays reported in the present study. **(a)** Dot plot showing the region gated corresponds to sperm. **(b)** Events gated in **(a)** are now plotted against SSC-H and SSC-A for select single cells, and this region was further used to set the remaining populations of interest. **(c)** Unstained control for viability and apoptosis. **(d)** Representative dot plot confronting Zombie Violet™ (X axis) versus CellEvent™ Caspase-3/7 Green fluorescence (Y axis), which evidences three subpopulations: (1) viable non-apoptotic sperm negative for Zombie Violet™ and CellEvent™ Caspase-3/7 Green, (2) viable apoptotic sperm negative for Zombie Violet™ and positive for CellEvent™ Caspase-3/7 Green (subpopulation of interest), and (3) dead sperm positive for Zombie Violet™ and CellEvent™ Caspase-3/7 Green. **(e)** Unstained control for viability and mitochondrial functionality. **(f)** Representative cytogram showing relation between Zombie Violet™ (X axis) and CellROX™ Deep Red (Y axis) which allowed us to detect three subpopulations: (1) viable sperm with low mitochondrial activity negative for Zombie Violet™ and CellROX™ Deep Red, (2) viable sperm with high mitochondrial activity negative for Zombie Violet™ and positive for CellROX™ Deep Red (subpopulation of interest), and (3) dead sperm positive for Zombie Violet™ and CellROX™ Deep Red.
